# Author Correction: A subterranean adaptive radiation of amphipods in Europe

**DOI:** 10.1038/s41467-022-30510-5

**Published:** 2022-05-12

**Authors:** Špela Borko, Peter Trontelj, Ole Seehausen, Ajda Moškrič, Cene Fišer

**Affiliations:** 1 SubBio Lab, Biotechnical Faculty, University of Ljubljana, Ljubljana, Slovenia; 2 Aquatic Ecology and Evolution, Institute of Ecology and Evolution, University of Bern, Bern, Switzerland; 3 Department of Fish Ecology and Evolution, Centre for Ecology, Evolution, and Biogeochemistry, Swiss Federal Institute of Aquatic Science and Technology (EAWAG), Kastanienbaum, Switzerland; 4 Agricultural institute of Slovenia, Ljubljana, Slovenia

**Keywords:** Biodiversity, Biogeography, Evolutionary ecology, Phylogenetics, Adaptive radiation

Correction to: *Nature Communications* 10.1038/s41467-021-24023-w, published online 17 June 2021.

The original version of this Article contained an error in Ref. 70, which incorrectly gave the reference as: Brikiatis, L. Late Mesozic North Atlantic land bridges. *Earth-Sci. Rev*. **159**, 47–57 (2016). The correct reference for Ref. 70 is: Brikiatis, L. The De Geer, Thulean and Beringia routes: key concepts for understanding early Cenozoic biogeography. *J. Biogeogr*. **41**: 1036-1054 (2014). This has been corrected in the PDF and HTML versions of the Article.

