# Supplementary material for: A subterranean adaptive radiation of amphipods in Europe
Source: Nat Commun. 2021 Jun 17;12:3688. doi: 10.1038/s41467-021-24023-w (PMC8211712; doi:10.1038/s41467-021-24023-w)
Supplement: Supplementary file 1 — Supplementary Information [file 41467_2021_24023_MOESM1_ESM.pdf]

A subterranean adaptive radiation of amphipods in Europe

SUPPLEMENTARY INFORMATION

Supplementary information includes:

Supplementary Figures

1) Chronogram (BEAST 2),

2) Ancestral Habitat Reconstruction (SIMMAP),

3) Node Height Test,

4) Geographical distribution of clades,

5) SURFACE Analysis,

6) Phylogram (IQTree),

7) Phylogram (MrBayes),

8) Chronogram of subset,

9) PCA plot.

Supplementary Tables

1) The results of the node height tests for each trait,

2) Calibration Comparisons,

3) PCA Summary,

4) List of amplification primers and PCR conditions,

5) Optimal substitution models selected by Partition Finder 2,

6) Calibration points used in BEAST 2.

Supplementary References

Supplementary Figure 1: Chronogram, inferred in BEAST2

Posterior probability higher than 0.5 is reported.

Blue bars represent 95 Highest Posterior Density interval for node ages.
Red arrows indicate calibration points.

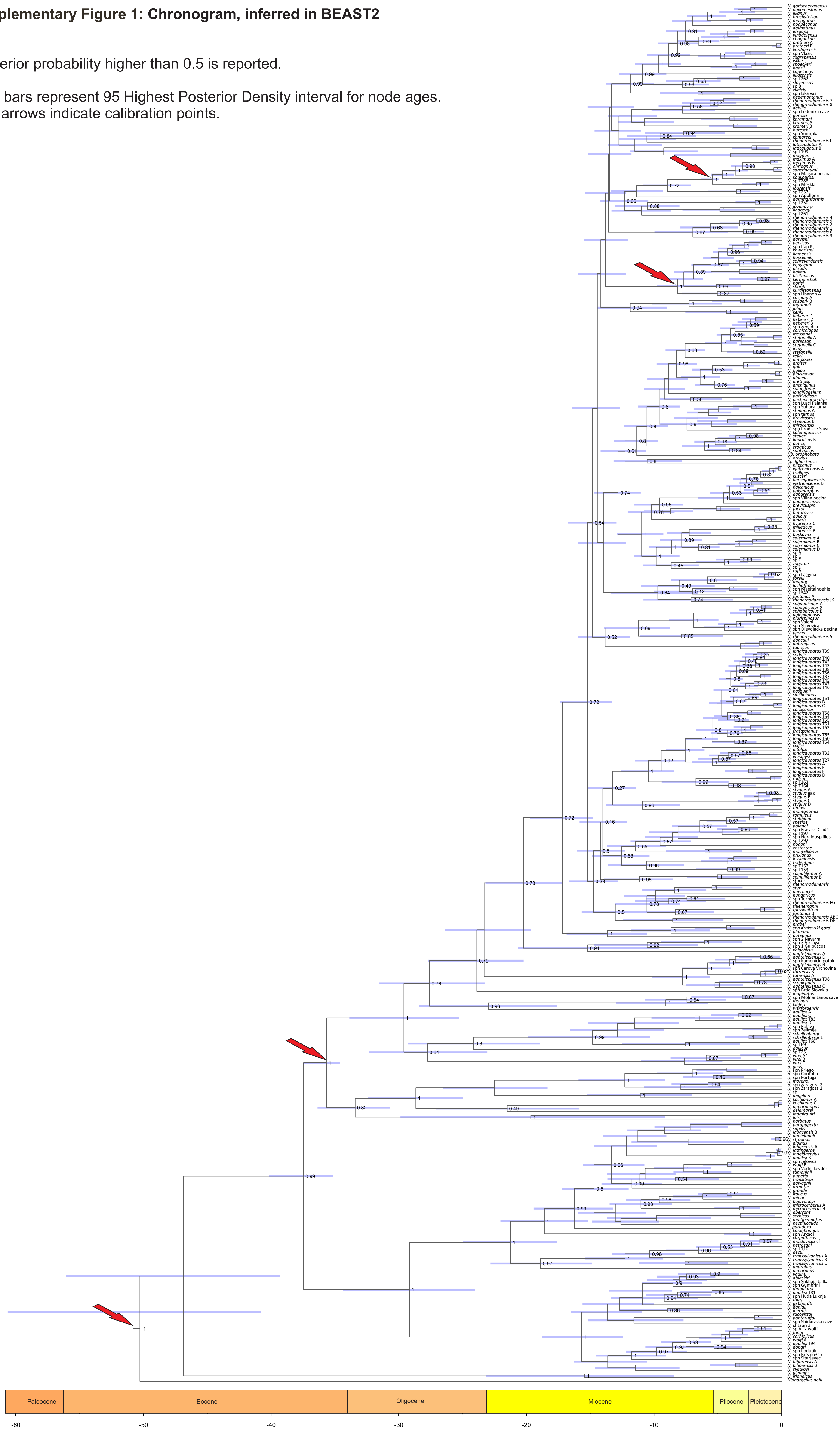

Supplementary Figure 2: Reconstruction of ancestral habitats

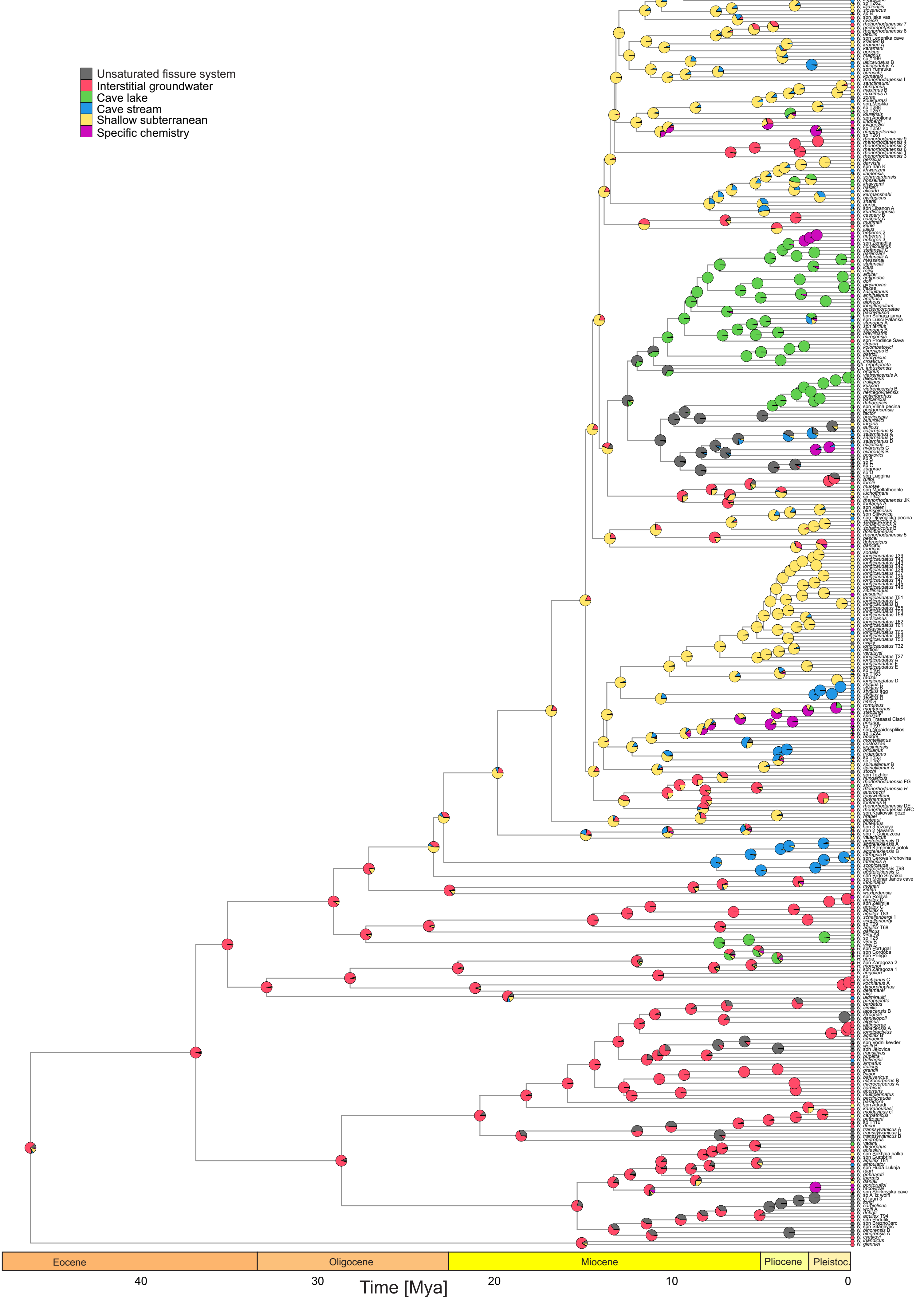

Supplementary Figure 3: Node height test. Scatter plots of standardized independent contrasts and the height above the root of the node for 11 morphological traits. Significant correlation indicates that the rate of trait evolution is changing systematically through the tree (see Supplementary Table 1 for results of linear model fits for each trait).

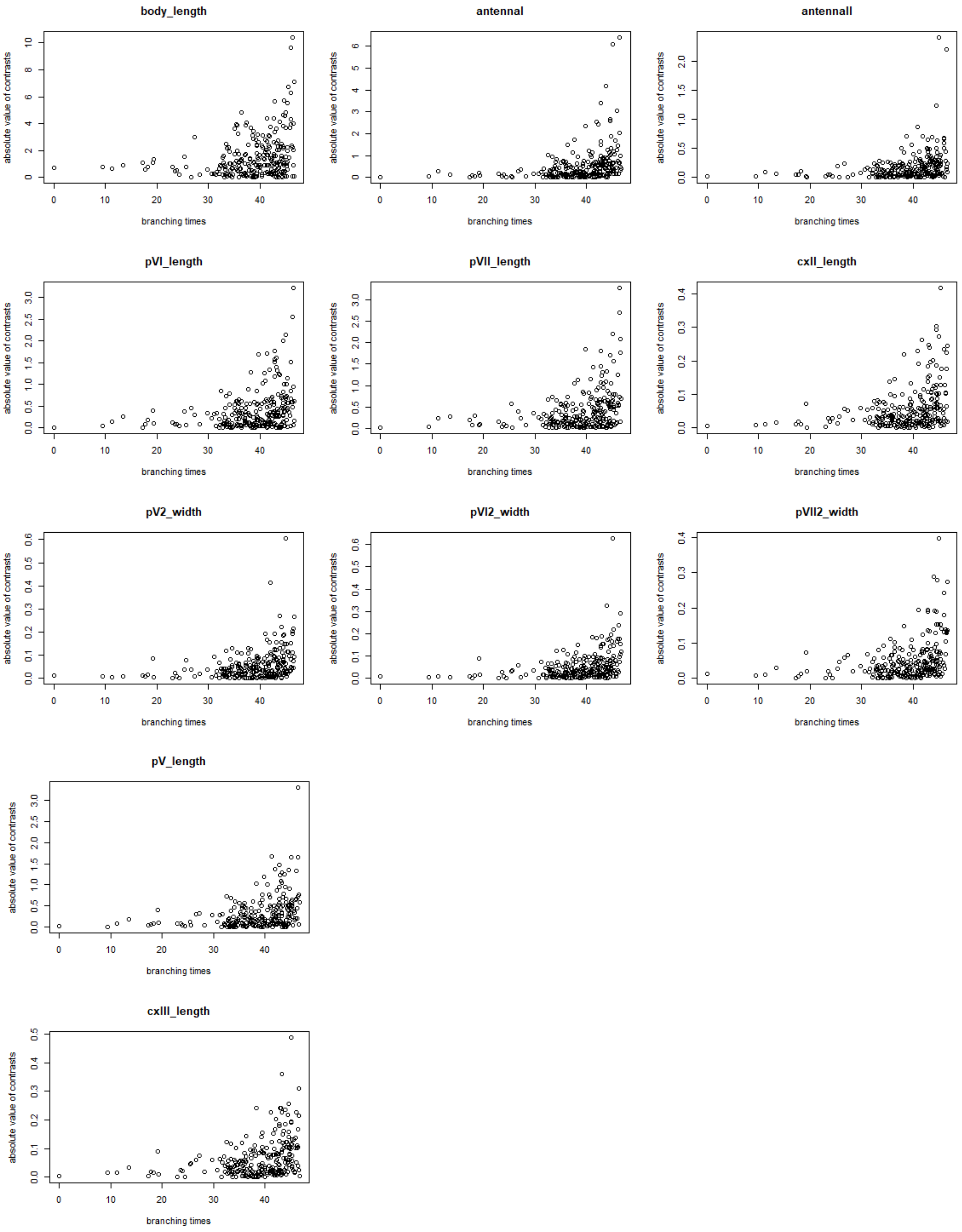

Supplementary Figure 4: Distributions of clades of interest, SubBioDatabase.
Maps were produced using QGIS[76] and Esri World Physical Map[77].

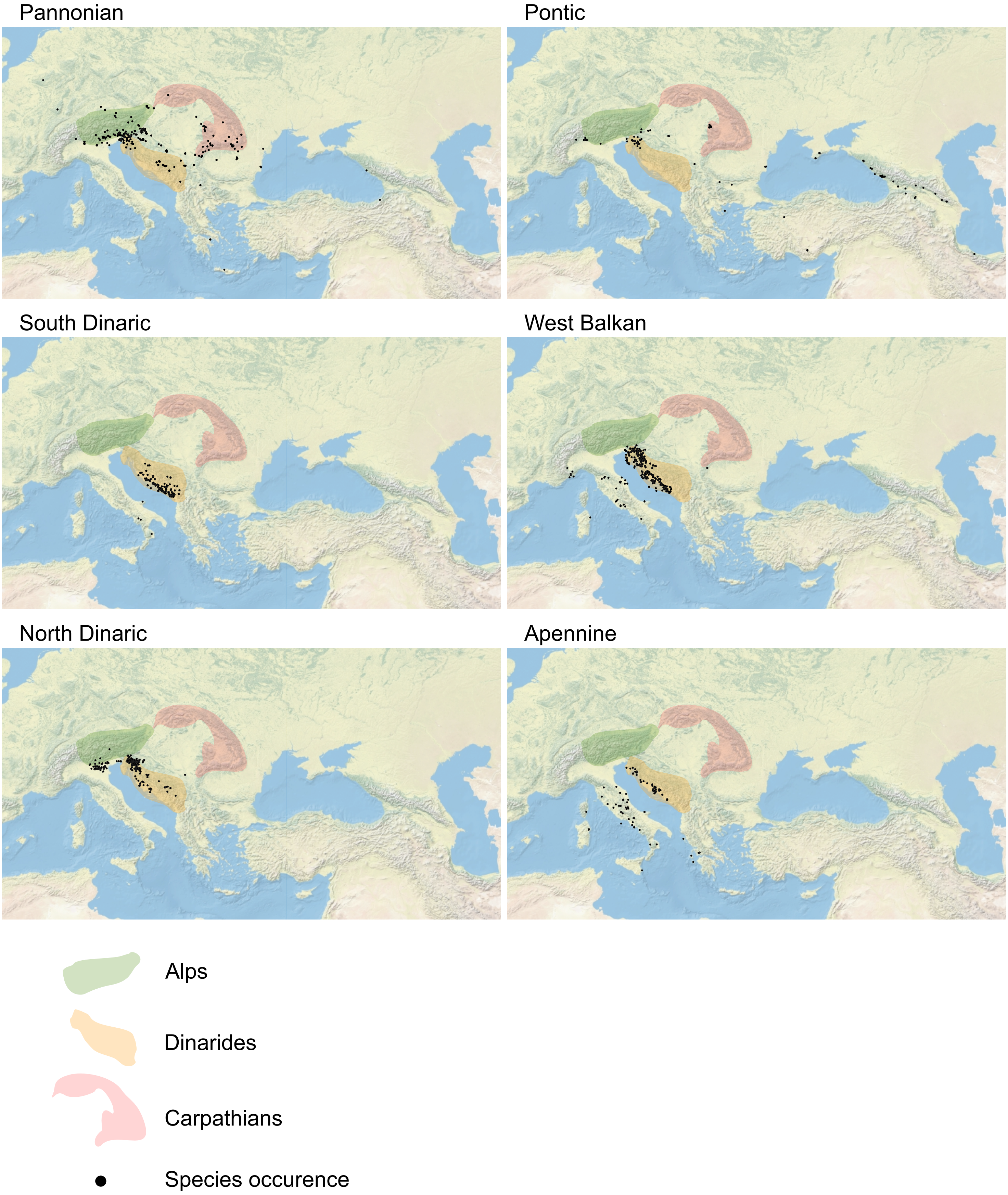

Supplementary Figure 5: SURFACE analysis, plotted on phylogeny. The best model predicted 14 different adaptive peaks and altogether 45 shifts. 11 peaks were convergent and 3 were not. Species from Pontic and Pannonian clades are generally small-pore inhabitants and on average an order of magnitude smaller than those of other clades. Relative morphological variation within these two clades is smaller as compared to variation in clades composed of larger species. To account for a potential lack of sensitivity, we repeated the analysis on a pruned tree, composed of only two clades. Indeed, this analysis identified additional adaptive peaks.

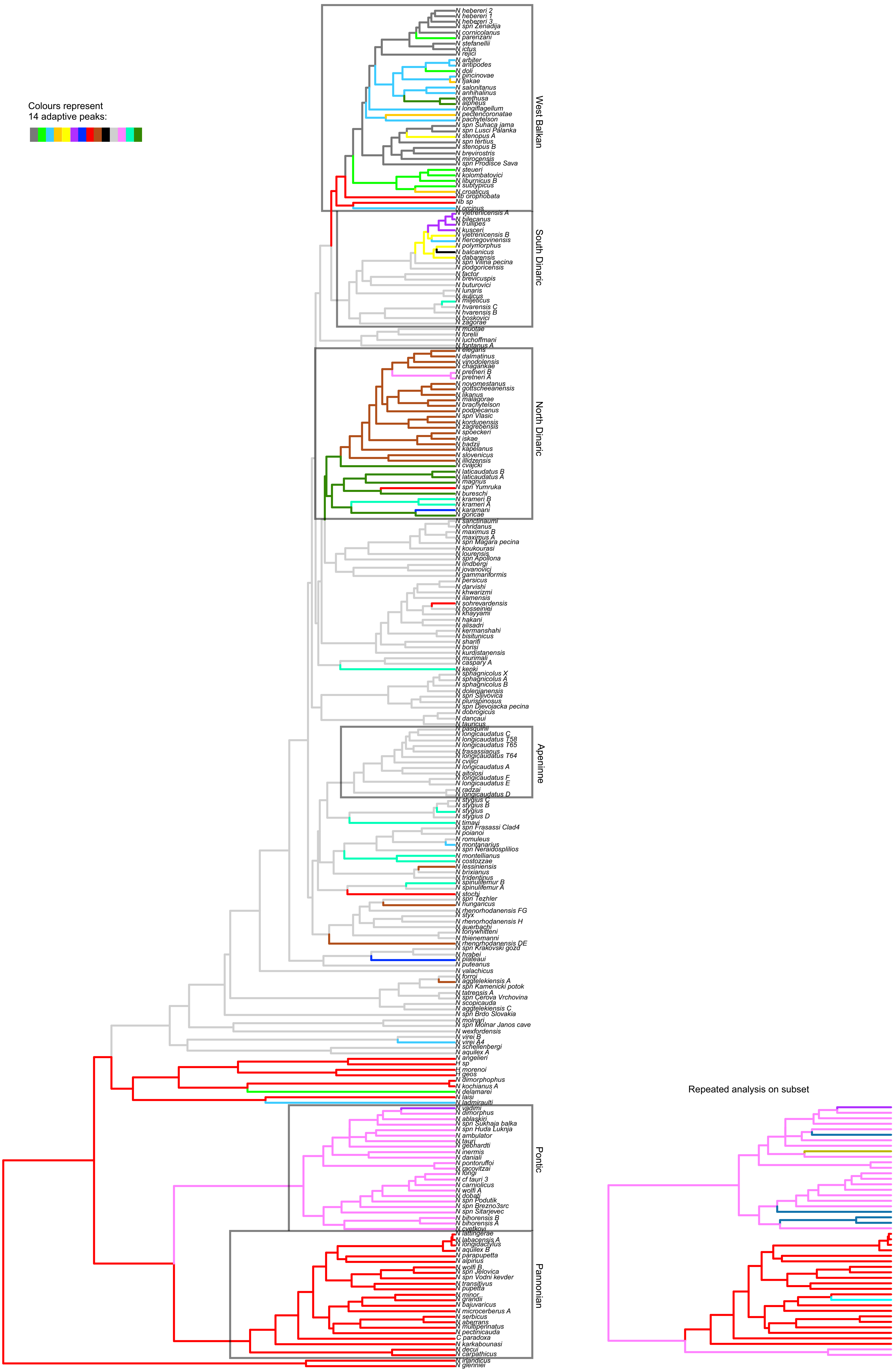

Supplementary Figure 6: Maximum Likelihood (IQTree)

301 *Niphargus* MOTUs and 5 outgroup MOTUs
Marked nodes:
SH-aLRT support (%) / ultrafast bootstrap support (%) > 80

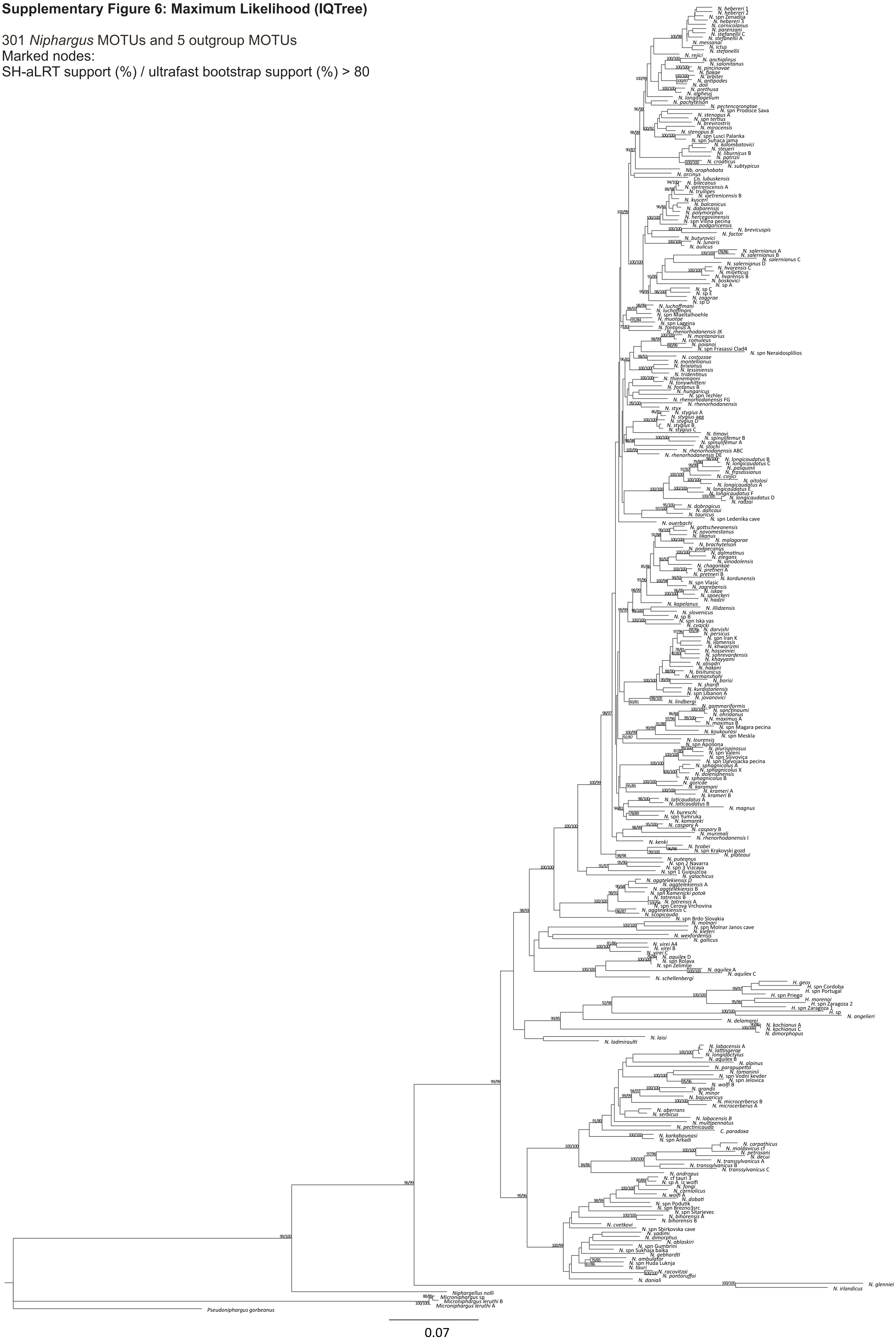

Supplementary Figure 7: Bayesian Inference (MrBayes)

301 *Niphargus* MOTUs and 5 outgroup MOTUs
Marked nodes: Posterior Probability > 80

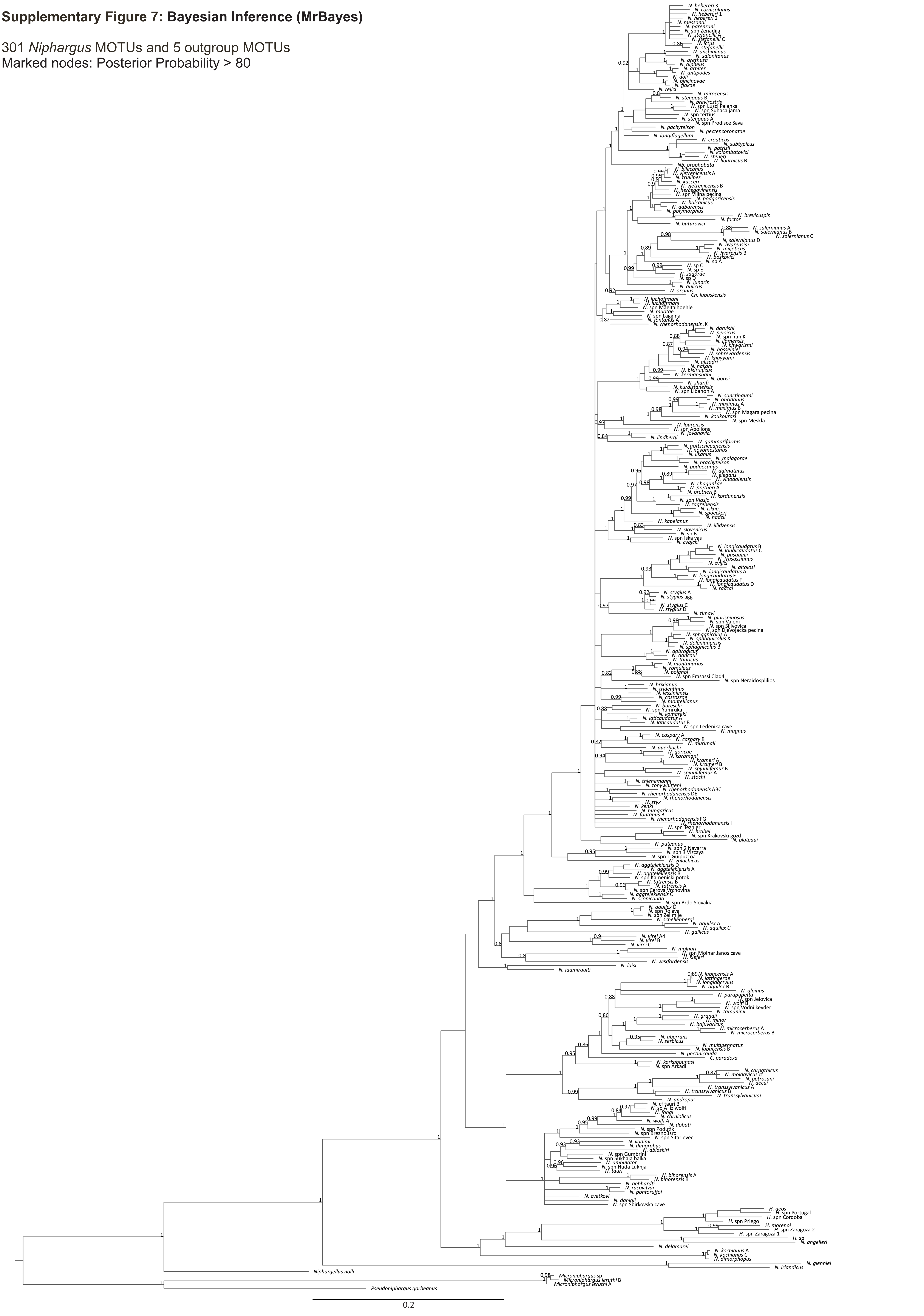

Supplementary Figure 8: Repeated analyses on subset of MOTUs with at least two markers (301 *Niphargus* MOTUs)

a) Chronogram, inferred in BEAST2

Posterior probability higher than 0.5 is reported.
Blue bars represent 95 Highest Posterior Density interval for node ages.
Red arrows indicate calibration points.

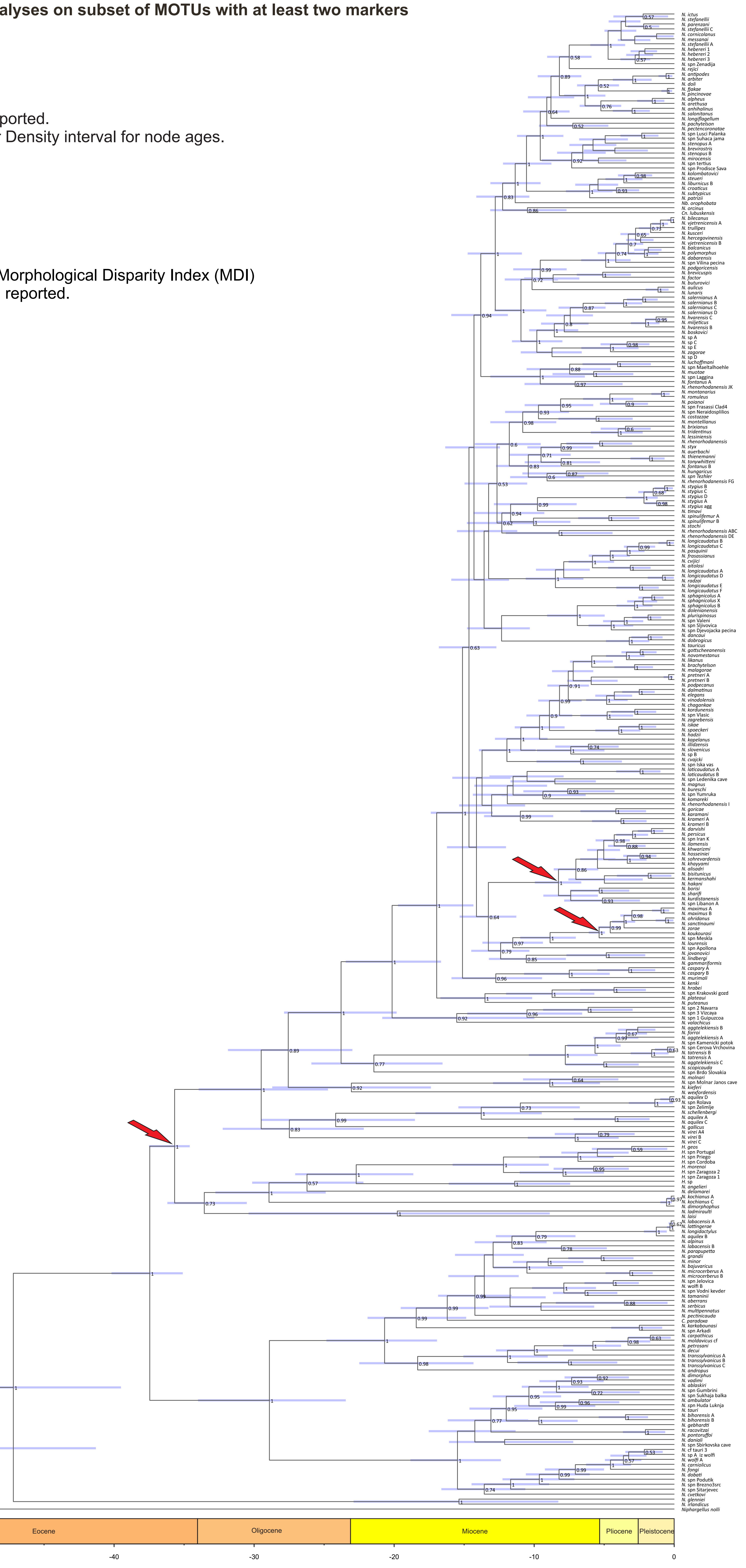

b) Through Time Plots

Pybus & Harvey's "gamma" statistic (γ), Morphological Disparity Index (MDI) and Ranked Envelope Test p-interval are reported.

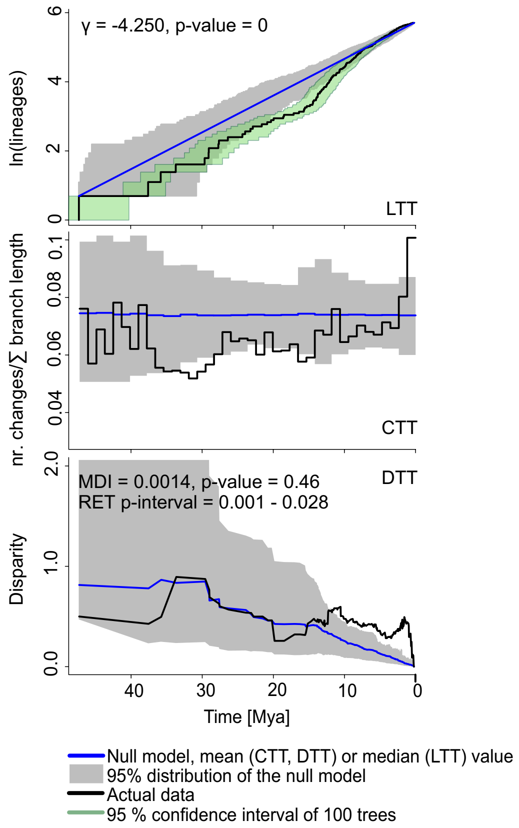

Supplementary Figure 9: Plot of Phylogenetically corrected PCA, inferred from raw values of eleven morphological traits. First two axes cover 97.5% of variability and are explained by body, antennae and leg length. Values are coloured by clusters, obtained from hierarchical Ward's clustering of the same traits, regressed onto body length (see Fig. 2C). Clustering identified several morphological types (Fig. 1). The relationship between morphology and habitat occupancy is imperfect, but relatively well expressed in synoptic populations[26]. Species (<10 mm) that live in fissure system (habitat category 1) are small with appendages of variable length; interstitial species (habitat category 2) can be divided into slender-long-legged and stout-short-legged ones (interstitial slender and interstitial stout in Fig. 1). Phreatic lakes (habitat category 3) harbour three morphotypes, all long legged: cave lake (large 15-23 mm), lake giants (>25 mm) and daddy-longlegs (large >20 mm, appendages longer than body length). Stream morphotypes, from two size classes, moderately large (10-15 mm) to large (15-25 mm) with short appendages, inhabit cave streams and shallow subterranean (habitat categories 4 and 5) and cannot be told apart by morphology. Chemical properties of habitat have no effect on herein studied morphological traits. Clusters are well reflected also on PCA plot, although some overlap is present. Note that clusters that are least distinguished by PCA are *Small pore stout, short appendages* and *Small pore slender, short appendages*, that partially also overlap with *Undistinct* cluster. Slenderness or stoutness is represented by depths of coxal plates and pereopod bases, which explain the fourth PCA axis; and undistinct morphotype is a generalist one, that falls in between other categories and can be found in a variety of habitats.

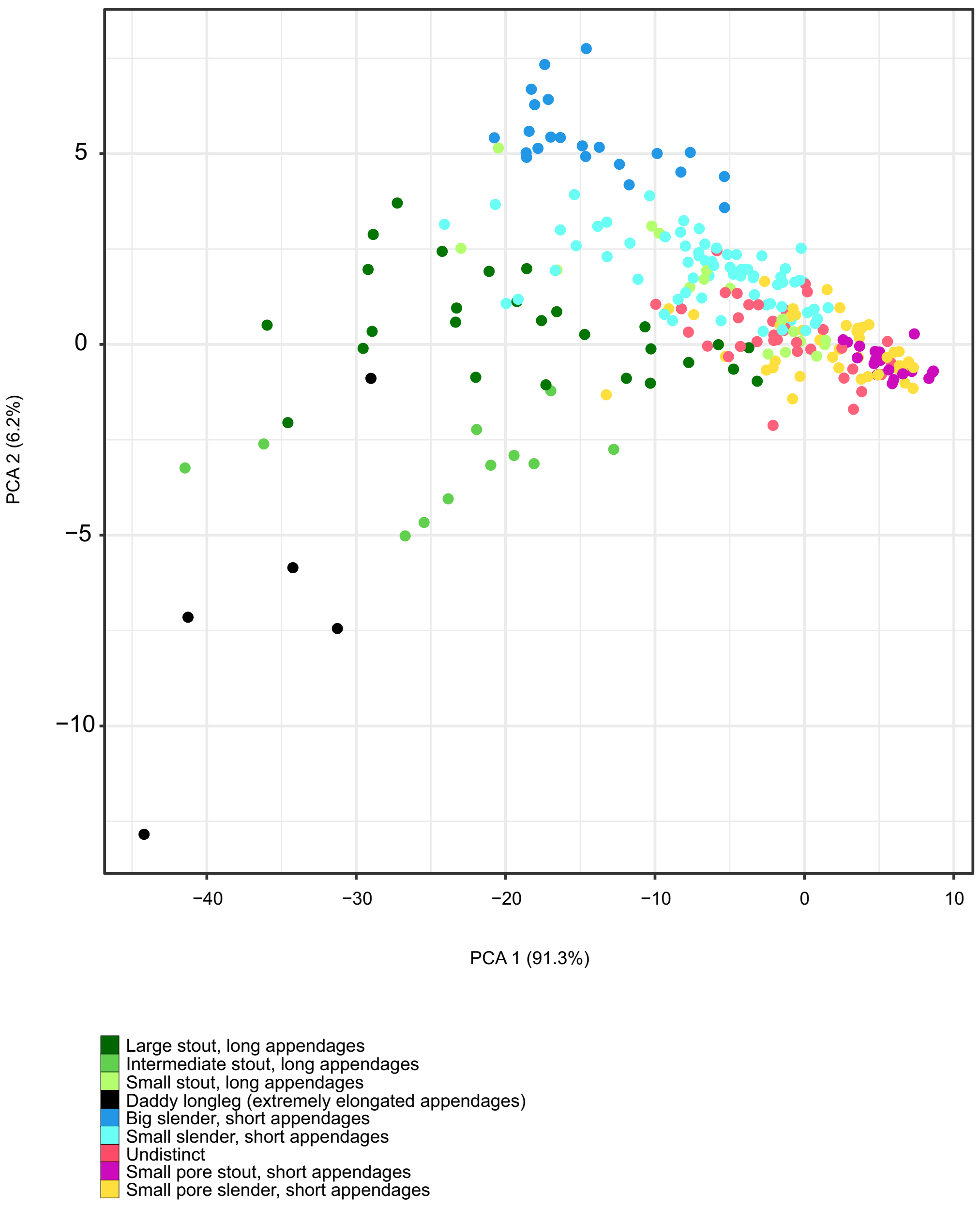

Supplementary Tables

Supplementary Table 1 The results of the node height tests for each trait.

trait	Adjusted R^2	p-value
Body length	0.07	8.48E-06
antennaI	0.09	7.19E-07
antennaII	0.08	3.95E-06
pV length	0.09	6.92E-07
pVI length	0.09	7.10E-07
pVII length	0.10	1.23E-07
cxII length	0.11	7.30E-08
cxIII length	0.11	4.10E-08
pV2 width	0.09	9.60E-07
pVI2 width	0.10	2.10E-07
pVII2 width	0.10	1.30E-07

The node height test fits a linear model between the absolute magnitude of the standardized independent contrasts and the height above the nodes for which the comparisons are made. The test was performed for each trait separately. Significant correlation indicates that the rate of trait evolution is changing systematically through the tree. Adjusted R^2 and p-value of two sided F-statistic are reported.

Supplementary Table 2 Comparison of phylogenetic reconstructions with different calibration points.

	Used calibration points				
**Node: Middle East**	**ALL**	**CRETE**	**MIDDLE EAST**	**FOSSIL**	**OUTGROUP**
Minimum	5.21	3.18	2.26	6.08	3.72
5% HPD	6.41	3.81	5.54	6.79	4.41
Median height	7.83	5.04	8.66	9.06	7.07
Mean height	7.85	5.10	8.64	9.34	7.50
95% HPD	9.40	6.53	11.83	12.43	11.49
Maximum	11.20	8.28	14.00	45.53	39.00
**Node: Crete**	**ALL**	**CRETE**	**MIDDLE EAST**	**FOSSIL**	**OUTGROUP**
Minimum	4.95	4.95	1.84	5.49	3.58
5% HPD	4.97	4.97	4.82	6.48	4.23
Median height	5.25	5.11	8.59	9.21	6.99
Mean height	5.35	5.14	8.71	9.47	7.42
95% HPD	6.00	5.40	12.57	12.60	11.31
Maximum	8.61	6.74	21.54	31.54	42.49
**Node: Fossil**	**ALL**	**CRETE**	**MIDDLE EAST**	**FOSSIL**	**OUTGROUP**
Minimum	25.79	12.65	9.31	34.61	16.42
5% HPD	29.63	14.77	20.53	34.61	18.83
Median height	33.01	18.98	34.42	36.91	29.06
Mean height	32.92	19.19	34.83	38.73	30.85
95% HPD	35.95	23.25	49.74	48.59	46.50
Maximum	40.11	29.85	64.05	149.29	120.75
**Node: European Niphargids**	**ALL**	**CRETE**	**MIDDLE EAST**	**FOSSIL**	**OUTGROUP**
Minimum	37.03	19.29	13.47	38.12	35.28
5% HPD	40.83	23.22	28.40	43.14	35.54
Median height	49.74	32.15	52.95	56.99	44.74
Mean height	50.28	32.52	54.10	59.01	47.92
95% HPD	60.61	41.93	79.21	79.51	68.58
Maximum	76.63	57.51	118.75	219.10	249.56

Five different phylogenetic reconstructions were calculated, using each calibration point separately and all four together. Summary of age reconstructions for four nodes at which calibration points were assigned is reported: minimum, maximum, 95% highest posterior density interval, and mean and median heights are reported for each node and each reconstructions.

Supplementary Table 3 Summary of phylogenetically corrected PCA.

	PC1	PC2	PC3	PC4	PC5	PC6	PC7	PC8	PC9	PC10	PC11
Standard deviation	3.39746	0.884014	0.436314	0.25006	0.173848	0.133538	0.088	0.071855	0.044633	0.037659	2.31E-02
Proportion of variance	0.913045	0.061816	0.015059	0.004946	0.002391	0.001411	0.000613	0.000408	0.000158	0.000112	4.21E-05
Cumulative proportion	0.913045	0.974861	0.98992	0.994866	0.997257	0.998667	0.99928	0.999688	0.999846	0.999958	1.00E+00

Standard deviation, proportion of variance and cumulative proportion are reported for 11 PC axes.

Supplementary Table 4 List of amplification primers and PCR conditions used.

Marker	Primers: name, sequence and source	PCR conditions described in
ArgK	ArgKin_F3 (CCCCTTCAACCCYTGYCTBACYGAGGC) and ArgKin_R3 (GGVAGCTTRATRTGGACGGAGGC)[1]	[1]
COI	LCO 1490 (GGTCAACAAATCATAAAGATATTG) and HCO 2198 (TAAACTTCAGGGTGACCAAAAAAT)[2]	[3]
EPRS	EPRS_1F (CAGGAAACAGCTATGACCGARAARGARAARTTYGC) and EPRS_1R (TGTAAAACGACGGCCAGTTCCCARTGRTTRAAYTTCCA)[4]	[1]
H3	H3AF2 (ATGGCTCGGTACCAAGCAGAC) and H3AR2 (ATRTCCTTGGGCATGATTGTTAC)[5]	[6]
HSP70	HSP70F324 (GATCATCGCCAACGAYCAGGG) and HSP70R960 (CGCTTGAAYTCYTGGATGAAGT)[7]	[7]
PEPCK	PEPCK_F3 (GAGGGCTGGCTRGCMGARCAYATG) and PEPCK_R3 (GGMCGCATTGCRAAYGGRTCRTGCAT)[8]	[1]
18S	18SF (CCTAYCTGGTTGATCCTGCCAGT) and 18SR (TAATGATCCTTCCGCAGGTT)[9]	[9]
28S lev2des2	28Slev2 (CAAGTACCGGTGAGGGAAAGTT) and 28Sdes2 (GTTCACCATCTTTCGGGTC)[10]	[6]
28s lev3des5	28Slev3 (GCCCTTAAAATGGATGGCGCT) and 28Sdes5 (CCGCCGTTTACCCGCGCTT)[6]	[6]

Supplementary Table 5 Optimal substitution models selected by Partition Finder 2.

Partition names	#sites	Best model
18S	2109	GTR+I+G
28S22	723	GTR+I+G
28S35	1178	SYM+I+G
ArgK	408	SYM+I+G
COI	657	GTR+I+G
EPRS	402	SYM+I+G
H3	330	SYM+I+G
HSP70	627	SYM+I+G
PEPCK	633	GTR+I+G

Supplementary Table 6 Calibration points used in BEAST 2.

Calibration point	Mean time	Distribution
European niphargids	Mean 64 (29), offset 35, sigma 0.95 (95% 37.9-154)	Log normal
Niphargus fossil	Mean 45 (10.4), sigma 1.4, offset 34.6 (95% 34.9-95.3)	Log normal
Crete clade	Mean 5.15 (0.23), sigma 0.55, offset 4.92, (95% 4.99 do 5.50)	Log normal
Middle East clade	Mean 9.5, sigma 1.52, (95% 6.52 – 12.5)	Normal

Supplementary References

1. Moškrič, A. and Verovnik, R. Five nuclear protein-coding markers for establishing a robust phylogenetic framework of niphargid crustaceans (Niphargidae: Amphipoda) and new molecular sequence data. *Data in Brief* **25**, 104134 (2019).
2. Folmer O.M., Black M., Hoeh R., Lutz R., Vrijehoek R. DNA primers for amplification of mitochondrial cytochrome c oxidase subunit I from diverse metazoan invertebrates. *Molecular Marine Biology and Biotechnology* **5**, 304–313 (1994).
3. Švara, V., Delić, T., Rađa, T. & Fišer, C. Molecular phylogeny of *Niphargus boskovici* (Crustacea: Amphipoda) reveals a new species from epikarst. *Zootaxa* **3994**, 3, 354 (2015).
4. Audzijonyte, A., Daneliya, M. E, Mugue, N., Väinölä, R. Phylogeny of Paramysis (Crustacea: Mysida) and the origin of Ponto-Caspian endemic diversity: resolving power from nuclear protein coding genes. *Molecular Phylogenetics and Evolution* **46**, 738–759 (2008).
5. Colgan, D. J., Ponder, W. F., Eggler, P. E. Gastropod evolutionary rates and phylogenetic relationships assessed using partial 28S rDNA and histone H3 sequences. *Zoologica Scripta* **29**, 29–63 (2000).
6. Fišer, C., Zagmajster, M., Zakšek, V. Coevolution of life history traits and morphology in female subterranean amphipods. *Oikos* **122**, 770–778 (2013).
7. Colson-Proch, C., Morales, A., Hervant, F., Konecny, L., Moulin, C., & Douady, C. J. First cellular approach of the effects of global warming on groundwater organisms: a study of the HSP70 gene expression. *Cell Stress & Chaperones* **15**, 3, 259–270 (2010).
8. Regier, J. C., Shultz, J. W. Molecular phylogeny of the major arthropod groups indicates polyphyly of crustaceans and a new hypothesis for the origin of hexapods. *Molecular Biology and Evolution* **14**, 9, 902–913 (1997).
9. Englisch, U., Coleman, C. O. & Wägele, J. W. First observations on the phylogeny of the families Gammaridae, Crangonyctidae, Melitidae, Niphargidae, Megaluropidae and Oedicerotidae (Amphipoda, Crustacea), using small subunit rDNA gene sequences. *Journal of Natural History* **37**, 20, 2461–2486 (2003).

10. Verovnik, R., Sket, B., Trontelj, P. The colonization of Europe by the freshwater crustacean *Asellus aquaticus* (Crustacea: Isopoda) proceeded from ancient refugia and was directed by habitat connectivity. *Molecular Ecology* **14**, 14, 4355–4369 (2005).
