# Supplementary material for: A subterranean adaptive radiation of amphipods in Europe
Source: Nat Commun. 2021 Jun 17;12:3688. doi: 10.1038/s41467-021-24023-w (PMC8211712; doi:10.1038/s41467-021-24023-w)
Supplement: Supplementary file 3 — Description of Additional Supplementary Files [file 41467_2021_24023_MOESM3_ESM.pdf]

Description of Additional Supplementary Files

File Name: Supplementary Data 1
Description: List of MOTUs used in the analyses along with vouchers, coordinates, accession numbers and hyperlinks to GenBank for used markers. Newly obtained sequences have the prefix "New". Chimera sequences are marked with vouchers of chimeras in the "chimera" column.

File Name: Supplementary Data 2
Description: Ancestral area reconstruction for each node for 1000 iterations. MOTUs that correspond to each node are listed below the table.

File Name: Supplementary Data 3
Description: Legend: A) unsaturated fissure system, B) interstitial, C) lake, D) stream, E) shallow subterranean, G) brackish, sulfidic, acidic or mineral.

File Name: Supplementary Data 4
Description: Morphological data for 11 continuous traits.
