# Supplementary material for: A subterranean adaptive radiation of amphipods in Europe
Source: Nat Commun. 2021 Jun 17;12:3688. doi: 10.1038/s41467-021-24023-w (PMC8211712; doi:10.1038/s41467-021-24023-w)
Supplement: Supplementary file 5 — Reporting Summary [file 41467_2021_24023_MOESM5_ESM.pdf]

nature research

Corresponding author(s): Špela Borko
Last updated by author(s): May 21, 2021

Reporting Summary

Nature Research wishes to improve the reproducibility of the work that we publish. This form provides structure for consistency and transparency in reporting. For further information on Nature Research policies, see our Editorial Policies and the Editorial Policy Checklist.

Statistics

For all statistical analyses, confirm that the following items are present in the figure legend, table legend, main text, or Methods section.

n/a	Confirmed	
☐	☒	The exact sample size (*n*) for each experimental group/condition, given as a discrete number and unit of measurement
☒	☐	A statement on whether measurements were taken from distinct samples or whether the same sample was measured repeatedly
☐	☒	The statistical test(s) used AND whether they are one- or two-sided<br>*Only common tests should be described solely by name; describe more complex techniques in the Methods section.*
☒	☐	A description of all covariates tested
☒	☐	A description of any assumptions or corrections, such as tests of normality and adjustment for multiple comparisons
☐	☒	A full description of the statistical parameters including central tendency (e.g. means) or other basic estimates (e.g. regression coefficient) AND variation (e.g. standard deviation) or associated estimates of uncertainty (e.g. confidence intervals)
☐	☒	For null hypothesis testing, the test statistic (e.g. F, t, r) with confidence intervals, effect sizes, degrees of freedom and P value noted<br>*Give P values as exact values whenever suitable.*
☐	☒	For Bayesian analysis, information on the choice of priors and Markov chain Monte Carlo settings
☒	☐	For hierarchical and complex designs, identification of the appropriate level for tests and full reporting of outcomes
☒	☐	Estimates of effect sizes (e.g. Cohen's *d*, Pearson's *r*), indicating how they were calculated

Our web collection on statistics for biologists contains articles on many of the points above.

Software and code

Policy information about availability of computer code

Data collection	Morphometric traits were measured with CellB (Olympus, 2008).
Data analysis	Sequences were edited, assembled and aligned in Geneious 11.0.3, with MAFFT v7.388 plugin. Phylogenies were obtained using Partition Finder 2, MrBayes 3.2.656, IQ-TREE 1.6.6 and BEAST 2. All analyses were run in R v.3.6.069, using packages vegan v.2.5-5, phytools v.0.6-60, geiger v.2.0.6.1, DDD v.4.0, surface v.0.5, mvMorph v.1.1.0, readxl v.1.3.1, RColorBrewer v.1.1-2, ggplot2 v.3.3.3, grid v.4.0.3, gridExtra v.2.3, dplyr v.1.0.2 and plyr v.1.8.6. Ancestral areas were reconstructed using BayesTraits V3.0.1. All R code is available on Zenodo under doi: 10.5281/zenodo.4779097.

For manuscripts utilizing custom algorithms or software that are central to the research but not yet described in published literature, software must be made available to editors and reviewers. We strongly encourage code deposition in a community repository (e.g. GitHub). See the Nature Research guidelines for submitting code & software for further information.

Data

Policy information about availability of data

All manuscripts must include a data availability statement. This statement should provide the following information, where applicable:

- Accession codes, unique identifiers, or web links for publicly available datasets
- A list of figures that have associated raw data
- A description of any restrictions on data availability

Sequence data has been deposited in GenBank. Newly obtained sequences are available in GenBank under accession numbers MT191378 - MT192029, MZ270543 and MZ295224. Vouchers, GenBank accession numbers, morphometric data, ecological data, and spatial coordinates of samples are listed in Supplementary Data 1, 3 and 4. Alignments and settings for phylogenetic analyses are available on Zenodo (10.5281/zenodo.4779097). European Groundwater Crustacean Database

(EGCD) used for compiling ecological information of species is accessible on http://data.freshwaterbiodiversity.eu/metadb/bf_mdb_view.php?uid=60abb94daccd5&code=35.

Field-specific reporting

Please select the one below that is the best fit for your research. If you are not sure, read the appropriate sections before making your selection.

☐ Life sciences ☐ Behavioural & social sciences ☒ Ecological, evolutionary & environmental sciences

For a reference copy of the document with all sections, see nature.com/documents/nr-reporting-summary-flat.pdf

Ecological, evolutionary & environmental sciences study design

All studies must disclose on these points even when the disclosure is negative.

Study description	The study analyses speciation and diversification patterns of amphipod genus Niphargus. We tested for adaptive radiation by modelling of lineage diversification and evolution of morphological and ecological traits using a time-calibrated multilocus phylogeny. The dataset is compiled of published GenBank sequence data, new sanger sequencing, morphometric traits and ecological data (newly obtained or gathered from literature) for 377 Niphargus MOTUs.
Research sample	The research sample consists of all described or not yet described Niphargus species with available molecular data, with rationale to cover as much of Niphargus diversity as possible. 377 Niphargus MOTUs are represented with one specimen on the phylogeny. The molecular data was newly obtained or gathered from GenBank. Ecological and morphometric data was newly obtained or gathered from European Groundwater Crustacean Database (EGCD).
Sampling strategy	Sample size covers all molecularly delimited Niphargus species, thus comprise the full range of ecological and taxonomic diversity of the genus. Each MOTU is represented with one sample. Morphological data and ecological obtained for as much species as possible (256 and 331 respectively). Measures of morphological traits were done on up to 20 adult individuals per species, or gathered from relevant literature when specimens not available.
Data collection	Amplification was done by Ajda Moškrič and Špela Borko, with help of Marjeta Konec. Sanger sequencing was done by Macrogen Europe laboratory (Amsterdam, Netherlands). Measuring the morphometric traits was done by Cene Fišer using a ColorView III camera mounted on an Olympus SZX stereomicroscope and the program CellB (Olympus, 2008); or collected from primary literature.
Timing and spatial scale	We utilized preserved samples from the collection of Subterranean Biology Laboratory, Biotechnical Faculty, University of Ljubljana, collected over past 20 years.
Data exclusions	No data was excluded from analyses.
Reproducibility	No experiment was conducted in this study.
Randomization	The work was not experimental in nature, and did not necessitate random assignment of specimens to treatment groups.
Blinding	Blinding was not relevant to our study. We collected all available data for amphipod genus Niphargus. To obtain morphometric data we measured adult specimens. Since subterranean organisms are scarce and hard to find in most cases we measured all available specimens, or we choose random 20 specimens. Blinding was not relevant for data analysis, we used all available data.

Did the study involve field work? ☐ Yes ☒ No

Reporting for specific materials, systems and methods

We require information from authors about some types of materials, experimental systems and methods used in many studies. Here, indicate whether each material, system or method listed is relevant to your study. If you are not sure if a list item applies to your research, read the appropriate section before selecting a response.

Materials & experimental systems

n/a	Involved in the study	
☒	☐	Antibodies
☒	☐	Eukaryotic cell lines
☒	☐	Palaeontology and archaeology
☐	☒	Animals and other organisms
☒	☐	Human research participants
☒	☐	Clinical data
☒	☐	Dual use research of concern

Methods

n/a	Involved in the study	
☒	☐	ChIP-seq
☒	☐	Flow cytometry
☒	☐	MRI-based neuroimaging

Animals and other organisms

Policy information about studies involving animals; ARRIVE guidelines recommended for reporting animal research

Laboratory animals	No laboratory animals were used in this work.
Wild animals	We utilized preserved samples of amphipodes (preserved in 70% or 95% ethanol) from the collection of Subterranean Biology Laboratory, Biotechnical Faculty, University of Ljubljana, collected over past 20 years. No additional capturing wild animals was performed for this study.
Field-collected samples	Study did not involved samples, collected in the field.
Ethics oversight	No ethical approval was required. No experiment on live animals was performed in this study. Vertebrates or higher invertebrates were not used in this study.

Note that full information on the approval of the study protocol must also be provided in the manuscript.
